# A comparison between physical therapy clinics with high and low rehabilitation volumes of patients with ACL reconstruction

**DOI:** 10.1186/s13018-023-04304-4

**Published:** 2023-11-07

**Authors:** Rebecca Simonsson, Johan Högberg, Jakob Lindskog, Ramana Piussi, Axel Sundberg, Mikael Sansone, Kristian Samuelsson, Roland Thomeé, Eric Hamrin Senorski

**Affiliations:** 1 Sportrehab Sports Medicine Clinic, Stampgatan 14, 411 01 Gothenburg, Sweden; 2 Sahlgrenska Sports Medicine Center, Gothenburg, Sweden; 3 Department of Orthopaedics, Institute of Clinical Sciences, Sahlgrenska Academy, University of Gothenburg, Gothenburg, Sweden; 4 Unit of Physiotherapy, Department of Health and Rehabilitation, Institute of Neuroscience and Physiology, Sahlgrenska Academy, University of Gothenburg, Box 455, 405 30 Gothenburg, Sweden; 5 Capio Ortho Center Gothenburg, Drakegatan 7A, SE-412 50 Gothenburg, Sweden; 6 Swedish Olympic Committee, Stockholm, Sweden; 7 Department of Orthopaedics, Sahlgrenska University Hospital, Mölndal, Sweden

**Keywords:** ACL, Reinjury, Knee, Sports, Physiotherapy, High/low volume

## Abstract

**Background:**

Treatment volume can impact outcomes after surgical procedures of the knee between surgeons with high- and low-patient-volumes. However, the difference between physical therapeutic clinics with high- and low-volumes has not been widely researched. This registry study aims to investigate how patient volume affects knee function outcomes after anterior cruciate ligament (ACL) reconstruction at physical therapy (PT) clinics in terms of odds for a second ACL injury, return to pre-injury level of activity, perceived knee function, and recovery of strength and hop performance.

**Method:**

Data were extracted from the Project ACL, a local rehabilitation registry. High- and low-volume clinics were defined based on the number of patients who attended different clinics. High-volume clinics were defined as those with > 100 patient registrations in Project ACL during the study period while low-volume clinics were those with ≤ 100 patient registrations. High- and low-volume clinics were compared, based on muscle function and patient-reported outcomes across 4 follow-ups, 2-, 4-, 8-, and 12 months, during the first year after ACL reconstruction, and odds of second ACL injury up to 2 years after ACL reconstruction.

**Result:**

Of the 115 rehabilitation clinics included, 111 were classified as low-volume clinics and included 733 patients, and 4 as high-volume clinics which included 1221 patients. There were 31 (1.6%) second ACL injuries to the ipsilateral or contralateral side within the first 12 months and 68 (4.0%) within 2 years. No difference in the incidence of a second ACL injury, within 12 months follow-up odds ratio (OR) 0.95 [95% CI 0.46–1.97] or within 2 years follow-up OR 1.13 [95% CI 0.68–1.88], was found between high- and low-volume clinics. There were early (2 months) and non-clinically relevant differences in patient-reported outcomes (PROs) and physical activity levels early after ACL reconstruction in favor of high-volume clinics. One year after ACL reconstruction, no differences were observed between high- and low-volume clinics in terms of PROs, muscle function, and return to pre-injury level of activity.

**Conclusion:**

No clinically relevant difference in the incidence of secondary ACL injuries in patients who underwent rehabilitation after ACL reconstruction at high- or low-volume physical therapist clinics was found. In addition, no clinically relevant differences in outcomes were found during the first year in terms of patient-reported outcomes, recovery of muscle function, or return to pre-injury level of activity.

**Supplementary Information:**

The online version contains supplementary material available at 10.1186/s13018-023-04304-4.

## Introduction

Anterior cruciate ligament (ACL) rupture is a serious knee injury that most commonly occurs in athletes participating in sports involving pivoting, cutting, or jumping movements [1]. Upon suffering an ACL injury, the rate of return to sport (RTS) is low after rehabilitation, regardless treatment with or without ACL reconstruction [2–4] with failure rate as high as 23% [5]. To improve outcomes after ACL injury both short- and long-term [3, 5, 6], the management of patients with an ACL injury requires improvement.

Many previous studies have investigated factors affecting second ACL injury, such as muscle function including achieving > 90% limb symmetry index [7, 8], timing of RTS [9], psychological readiness of RTS [10, 11] and biomechanical movement patterns [12]. One factor that has not been widely researched is the difference between clinics with high- and low-patient volume. Treatment volumes have been shown to influence outcomes after surgical procedures of the knee, where the mean operating room time for performing ACL reconstruction or meniscectomy was significantly shorter in hospitals and surgeons with high patient volumes [13]. However, whether this shorter time leads to better treatment results is unknown. Intuitively, a higher patient volume may contribute to the development of expertise, thereby improving treatment outcomes. With regard to ACL rehabilitation, Grindem et al. [14] reported that patients undergoing rehabilitation in a specialized sports medicine center with more progressive rehabilitation can report superior patient-reported outcomes (PROs) compared to usual care 2 years after ACL reconstruction. However, the only outcome measured was knee function, reflected by the Knee injury and Osteoarthritis Outcome Score (KOOS), and the patient sample size for the sports medicine clinic group was low compared with the usual care group (84 vs. 2690). To deepen the understanding of the impact of physical therapy clinical volume we wanted to investigate the relationship between clinics with high- or low-patient volumes on functional and psychological outcomes, where high-volume clinics were used as a proxy for more experienced physical therapists (PTs).

The purpose of this study was to investigate the volume—outcome relationship with regard to the odds of a second ACL injury, return to the pre-injury level of activity, perceived knee function, and the recovery of strength and hop performance in patients undergoing rehabilitation at PT clinics with high- and low-patient volumes after ACL reconstruction.

## Materials and methods

### Design

This study was performed according to the REporting of studies Conducted using Observational Routinely Collected Health Data (RECORD) statement [15] using registry data. Data were collected from a local rehabilitation outcome registry for patients with ACL injuries (Project ACL; Gothenburg, Sweden). Project ACL aims to improve care after an ACL injury by regularly evaluating patients using standardized and validated tests for muscle strength, hop performance, and PROs. For patients enrolled in Project ACL, evaluations were scheduled after injury or within 6 weeks preoperatively with ACL injury or surgery as the baseline, and then at 10 weeks, 4-, 8-, 12-, 18-, and 24 months, 5 years, and then every 5 years after ACL injury/reconstruction (see Additional file 1 for follow-up procedure). Participation in the registry is voluntary and can be withdrawn at any time. Patients independently choose where to perform their rehabilitation, and rehabilitation programs are individualized. Informed consent was obtained from patients at time of registration in Project ACL. Ethical approval has been obtained from the Swedish Ethical Review Authority (registration number 2020-02501).

### Inclusion/exclusion

Patients participating in the Project ACL with data from their primary ACL reconstruction who had registered a clinic where they performed their rehabilitation were eligible for inclusion. Patients with autografts harvested from the contralateral side were excluded because the measurement of limb symmetry index would not have a “healthy” limb to represent the patient 100%.

### Data collection

Upon registration in Project ACL, patients were asked to provide information on their rehabilitation clinic and PT. The number of patients registered per clinic was calculated at the time of data collection. The definitions of high- and low-patient volume clinics were determined after assessing the distribution of data collected in Project ACL, by how many patients each clinic had registered at data extraction in November 2021. The definition of high-volume clinics was set at > 100 registrations, and ≤ 100 registrations for low volume clinics. Patient volumes were used as a proxy for clinical experience with rehabilitation of patients after ACL reconstruction. The cutoff of 100 was chosen based on visual assessment of the plotted number of registered patients during the study period, Additional file 2. To handle cases in which patients changed from a high-volume to a low-volume clinic, or vice versa, during their rehabilitation, the clinic where the patient had attended most follow-ups during the rehabilitation was used. Data on patient demographics, second ACL injuries, defined as re-rupture or contralateral ACL injury, which the patient him-/herself, the responsible physical therapist or the orthopedic surgeon manually had registered in the database, return to pre-injury level of activity, results from muscle function tests, and PROs were extracted from the Project ACL database.

### Test battery

The muscle function tests included in the Project ACL test battery consisted of validated muscle strength tests for unilateral knee extension and knee flexion, as well as unilateral hop tests: vertical hop, hop for distance, and 30 s side hop test. The tests were supervised by registered PTs trained in standardization of the tests. Strength test evaluation started 10 weeks after ACL reconstruction, whereas hop tests started 4 months after ACL reconstruction, with permission from the treating PTs. Prior to testing, the patients performed 10-min warm-up on a stationary bike.

Muscle strength was tested as a maximal isokinetic concentric strength test for knee extension and flexion using a seated dynamometer (Biodex System 4; Biodex Medical System, Shirley, NY, USA) with an angular speed of 90° per second [16]. The test started with a familiarization phase where patient performed 10 submaximal attempts at 50% and 75%, followed by 1–2 repetitions of 90% of their maximal capacity before starting the maximal trials. After familiarization, the patients performed three to four maximal attempts with a 40 s rest between each attempt. The highest result in Newton meters (Nm) of all attempts was registered in the Project ACL database.

In all hop tests, the patients were asked to hold their hands behind their backs. Patients were allowed 2–3 submaximal attempts before performing the tests. For the vertical hop test, the time from takeoff to landing was measured and converted to centimeters (cm) using the Muscle Lab (Ergotest Technology, Oslo, Norway) [17]. In the hop for distance test, patients were required to perform a stable landing, that is, landing on the jumping foot without releasing hands from behind the back, not moving the jumping foot, or supporting their balance with the opposite foot. The distance from the toes at takeoff to the heel at landing was measured in centimeters (cm). In the 30 s side hop, patients were instructed to jump as many times as possible past two lines 40 cm apart for 30 s. The hop tests used in Project ACL have been shown to have high reliability in patients who have undergone ACL reconstruction [17].

### Patient reported outcomes

#### Four PROs were used in this study

The Tegner Activity Scale (Tegner) was developed to assess the level of knee-strenuous physical activity [18]. The scale is graded from 0 to 10, where higher values indicate increased physical demands on the knee [18], 0 equals sick leave, and 10 equals American football or soccer on an elite level [19]. Project ACL uses a modified version of Tegner, which starts at Level 1. Tegner level 1–5 was used as a proxy for work and physical activity and defined Tegner level 6–10 as participation in knee-strenuous sports.

The ACL Return to Sport after Injury (ACL-RSI) is a 12-item scale, developed to measure the patients psychological readiness of RTS after ACL reconstruction [20]. The scale is graded from 1 to 10 for each item, where 1 reflects extremely negative psychological responses and 10 reflects extremely positive psychological responses prior to RTS [20]. The scale scoring used in Project ACL was modified to a maximum score of 120 points and a minimum score of 10, whereas the original scale ranges from 10 to 100.

The Knee Self-Efficacy Scale_18_ (K-SES_18_) is a reliable instrument that aims to measure perceived knee-related self-efficacy in patients with ACL injury [19]. In this study, the K-SES_18_ was used, which consists of 18 items divided into two subscales. The first subscale relates to the patient’s present knee self-efficacy consisting of 14 items, and the second subscale to future knee self-efficacy consisting 4 items. Each item is graded from 0 to 10. A score of 0 indicated poor self-efficacy and 10 indicates strong self-efficacy. An arithmetic mean was used for comparison between patient values [19].

The KOOS was developed to assess symptoms following knee injuries [21]. The KOOS consists of five subscales: pain, symptoms, activities of daily living, function in sports and recreation, and quality of life. Each subscale was analyzed separately, with a score of 0 reflecting severe symptoms and 100 indicating no symptoms [21].

### Primary outcomes

The primary outcome of this study was the 12 months, and 2-years incidence of second ACL injury, i.e., either graft rupture or contralateral ACL rupture, and the rate of return to the pre-injury level of activity, defined as returning to the same Tegner as pre-injury at 12 months follow-up.

### Secondary outcomes

The secondary outcomes were the rate of passing the muscle function tests included in the Project ACL, as well as the results of the muscle function tests and PROs during the first year after ACL reconstruction. Passing the muscle function test was defined as achieving ≥ 90% limb symmetry index (LSI), i.e., result from injured limb divided by result of the uninjured limb expressed as a percentage [22].

To assess the recovery of muscle function, only patients who participated in the muscle function tests at all scheduled follow-ups (2-, 4-, 8-, and 12 months) were included for this analysis. This enabled us to study a ‘compliant’ cohort of patients after ACL reconstruction. To assess the PROs, only patients who had answered to the questionnaires at all follow-ups were included for this analysis.

### Statistical analysis

Statistical analysis was performed using Statistical Product and Service Solutions (IBM SPSS Statistics for Windows, version 25.0. Armonk, NY, USA). Mean values were presented with standard deviations (SD), and medians with minimum and maximum values. All comparisons were made between high- and low-volume clinics. The alpha level was set to 0.05. For comparisons between high- and low-volume clinics, Fisher’s exact test was used for categorical variables, the Mann–Whitney *U*-test for nonparametric variables, and the independent *t*-test for continuous variables. The Levene’s test was used to determine equality of variance. To interpret the clinical relevance of significant findings, Cohen’s *d* (*d*) was used with the following reference values: 0.20 = small, 0.50 = medium, and 0.80 = large [23]. The odds of sustaining a second ACL injury, stratified by clinics with high- or low-patient volume, was presented as an odds ratio (OR) with 95% confidence interval (CI) for risk estimates not including 1.00. In addition, a sample size calculation based on a 5% difference in the main outcome (second ACL injury), expecting 80% power and a significance level of 0.05, suggested that 868 individuals were required for the study.

## Results

**Fig. 1:**
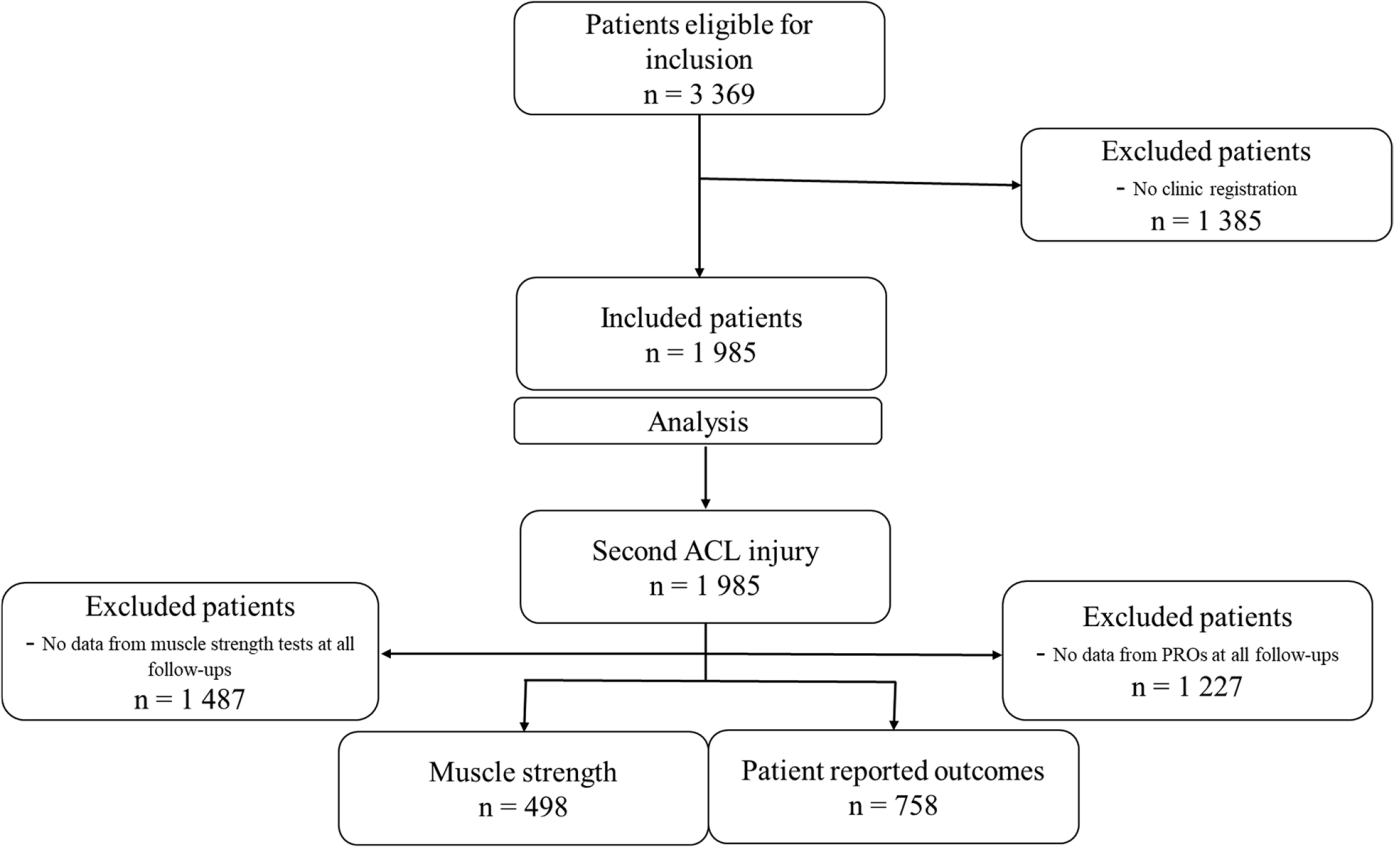
Inclusion/exclusion process and distribution of the cohort in different analysis

A total of 1985 patients were included, of which 498 had completed all muscle strength tests and 758 had completed all PROs during the first year (Fig. 1). There were four high-volume (> 100 patients) and 111 low-volume clinics, distribution is presented as Additional file 2.

In total, 31 (1.6%) second ACL injuries were sustained within 12 months after ACL reconstruction, with an additional 37 (2.4%) second ACL injuries sustained between 1 and 2 years after ACL reconstruction. Table 1 presents the odds ratios for the incidence of a second ACL injury within 1 or 2 years after ACL reconstruction. No significant differences were observed in this study. Full details of the second ACL injury rates stratified by age are available in Additional files 3 and 4.

**Table 1:** Odds ratio for sustaining a second ACL injury, by high- and low-patient volume clinics

	*n*	12 months incidence,* n* (%)	Odds ratio [CI]	*p*-value
Total	1985	31 (1.6%)	0.95 [0.46–1.97]	0.89
HV	1221	19 (1.5%)		
LV	733	12 (1.6%)		

	*n*	2 years incidence, *n* (%)	Odds ratio [CI]	*p*-value
Total	1686	68 (4.0%)	1.13 [0.68–1.88]	0.64
HV	1001	44 (4.4%)		
LV	617	24 (3.8%)		

*ACL* anterior cruciate ligament, *n* number of patients, *HV* high-volume clinics, *LV* low-volume clinics, *CI* confidence interval

### Return to pre-injury level of activity

**Fig. 2:**
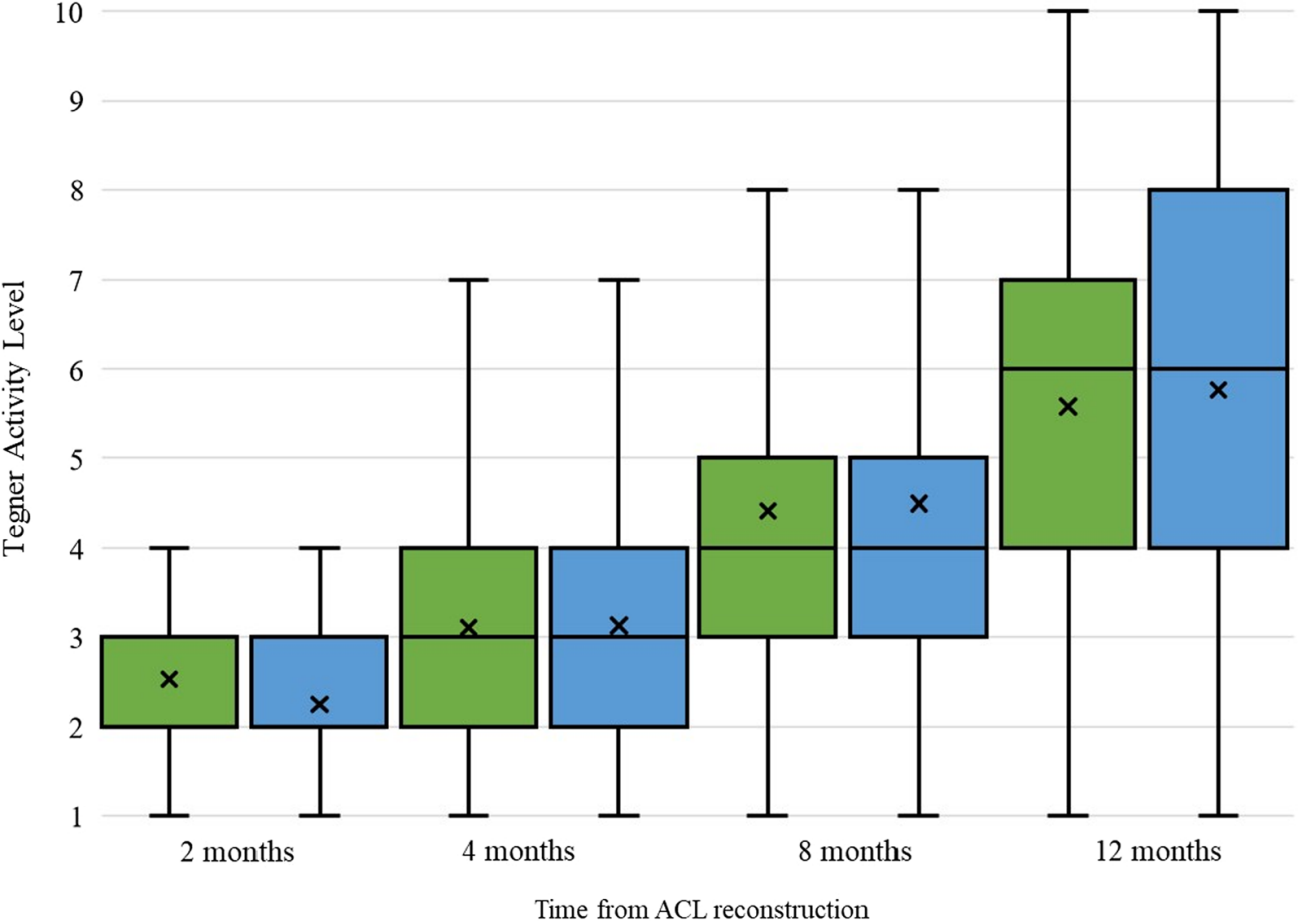
The change in physical activity level during the first 12 months after anterior cruciate ligament reconstruction and the rate of returning to pre-injury level of activity stratified by Tegner Activity Scale. Green and blue boxes represent high and low volume clinics, respectively. The x represents mean values, the line represents median values, which is 2 for first two boxes, and the whiskers represent minimum and maximum values

The rate of returning to the pre-injury level of activity in patients with a pre-injury Tegner score of 1–5 was 62% for high-volume (HV) and 71% for low-volume (LV) clinics at the 12 months follow-up. For patients who had a higher pre-injury level of activity (Tegner 6–10), the rate of return to the same activity level was 26% for HV and 31% for LV clinics at the 12 months follow-up. A greater proportion of patients returned to the pre-injury level of activity, at 2 and 4 months in the HV group than in the LV group given a pre-injury Tegner level of 1–5 (Fig. 2  and Table 2). No other differences were observed in the rate of return to the pre-injury level of activity regardless of the pre-injury Tegner level at any follow-up, up to 12 months after ACL reconstruction. Patients in the HV group had a significantly higher Tegner score at 2 months than those in the LV group (*p* = 0.03). No differences were observed at 4, 8, or 12 months at the Tegner level (Table 2).

**Table 2:** Change in Tegner and rate of returning to pre-injury Tegner, first 12 months after ACL-reconstruction

Return to pre-injury level of activity	2 months			4 months			8 months			12 months		
	HV (%)	LV (%)	*p*-value	HV (%)	LV (%)	*p*-value	HV (%)	LV (%)	p-value	HV (%)	LV (%)	*p*-value
Tegner 1–5, (%)	34	11	0.04	42	23	0.03	58	42	0.08	62	71	0.35
Tegner 6–10, (%)	5	2	0.05	4	5	0.69	9	11	0.58	26	31	0.20

### Patient-reported outcomes

A total of 758 patients had available data on PROs from all follow-ups during the first year after ACL reconstruction, of which 63% and 37% represented the HV and LV groups, respectively (Table 3). There were no significant differences in the demographics.

**Table 3:** Demographics for patients, with available PROs-data, in high- and low-volume clinics, first 12 months after ACL-reconstruction

	HV	LV	*P*-value
*n*	477	281	
Women, *n* (%)	274 (57.4%)	162 (57.5%)	1.00
Age (years)	31 ± 11	32 ± 11	0.42
Height (cm)	173 ± 9	174 ± 9	0.19
Weight (kg)	72 ± 13	73 ± 13	0.32
BMI (kg/m^2^)	23.8 ± 3.4	23.8 ± 3.2	0.99
Days between injury and reconstruction	269 ± 475	387 ± 908	0.05
Type of graft			
Hamstrings	375 (81%)	203 (80.8%)	0.88
Patella	75 (16.2%)	41 (16.4%)	
Allograft	4 (0.9%)	4 (1.6%)	
Quadriceps	3 (0.6%)	1 (0.4%)	
Other	6 (1.3%)	2 (0.8%)	
Tegner pre-injury, *n* (%)			
1	5 (1.0%)	1 (0.4%)	0.15
2	10 (2.1%)	4 (1.4%)	
3	13 (2.7%)	8 (2.8%)	
4	26 (5.5%)	22 (7.8%)	
5	22 (4.6%)	19 (6.8%)	
6	42 (8.8%)	33 (11.7%)	
7	89 (18.7%)	31 (11.0%)	
8	87 (18.2%)	57 (20.3%)	
9	127 (26.6%)	77 (27.4%)	
10	56 (11.7%)	29 (10.3%)	

*ACL* anterior cruciate ligament, *n* number of patients, *cm* centimeters, *kg* kilogram, *BMI* body mass index, *m* meters, *HV* high-volume clinic, *LV* low-volume clinic, *PROs* patient reported outcomes

For categorical variables, *n* (%) is presented

For continuous variables, the mean ± SD is presented

**Fig. 3:**
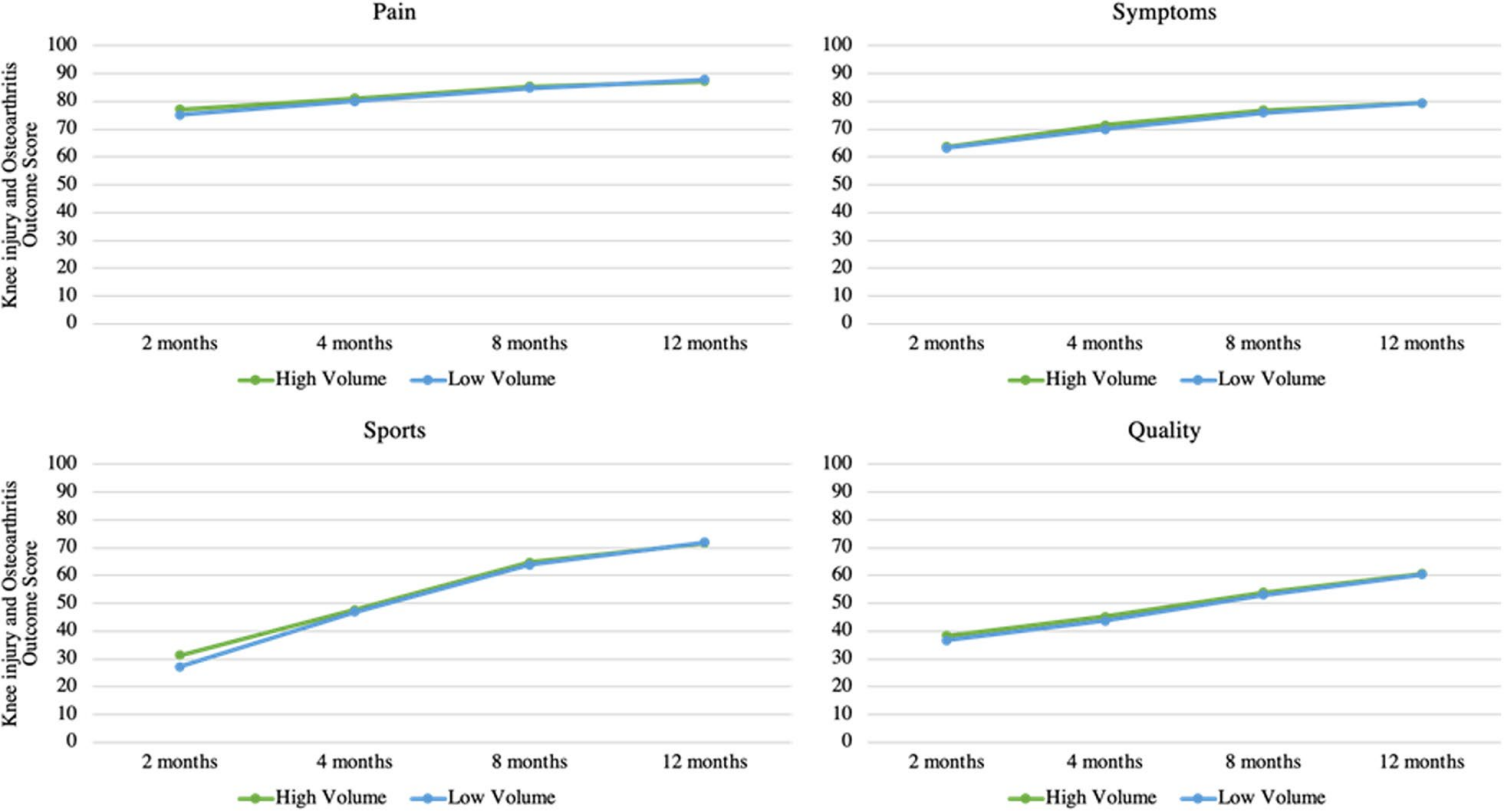
Subscales of Knee injury and Osteoarthritis Outcome Score for high and low volume clinics during the first 12 months after anterior cruciate ligament reconstruction

Patients treated at HV clinics reported a greater Knee injury and Osteoarthritis Outcome Score-Sports (KOOS-Sports) score at 2 months after ACL reconstruction, compared with patients treated at LV clinics, although the effect size was small (31.3 ± 22.1 HV vs. 27.2 ± 19.9 LV, *p* = 0.014, *d* = 0.186). The results from all KOOS subscales during the first year are shown in Fig. 3 and Table 4. There were no differences between the groups for any of the other PROs included in this study at any follow-up during the first year after ACL reconstruction. The values for K-SES_18_ and ACL-RSI are listed in Additional file 5.

**Table 4:** Subscales of the KOOS for high- and low-volume clinics, first 12 months after ACL-reconstruction

PROs	Group	2 months	4 months	8 months	12 months
KOOS-pain	HV	77.1 ± 14.2	81.1 ± 13.0	85.4 ± 12.6	87.3 ± 12.8
	LV	75.1 ± 14.1	80.0 ± 13.2	84.8 ± 12.0	87.9 ± 10.6
KOOS-symptom	HV	63.8 ± 17.3	71.5 ± 16.3	76.9 ± 16.3	79.5 ± 16.1
	LV	63.3 ± 17.3	70.0 ± 17.3	75.9 ± 16.1	79.3 ± 15.2
KOOS-sports	HV	31.3 ± 22.1*	47.7 ± 22.6	64.8 ± 21.9	71.7 ± 23.1
	LV	27.2 ± 19.9*	46.9 ± 21.8	63.9 ± 21.4	71.9 ± 21.4
KOOS-quality	HV	38.4 ± 16.2	45.2 ± 17.4	54.0 ± 18.9	60.7 ± 19.8
	LV	36.7 ± 15.6	43.7 ± 15.1	53.0 ± 18.3	60.4 ± 18.9

*ACL* anterior cruciate ligament, *HV* high volume, *KOOS* Knee injury and osteoarthritis outcome score, *LV* low volume, *PROs* Patient reported outcomes

*Significant (*p* < 0.05) difference between high- and low-volume clinics. All results are presented as mean ± SD

### Muscle strength

For muscle strength testing, 497 patients attended every follow-up during the first 12 months after ACL reconstruction, of which 68% and 32% represented the HV and LV groups, respectively (Table 5). No significant differences were observed in the demographic characteristics of this cohort.

**Table 5:** Demographics for patients attending muscle strength testing at all follow-ups, first 12 months after ACL-reconstruction

	HV	LV	*p*-value
*n*	338	159	
Women, *n* (%)	182 (53.8%)	90 (56.6%)	0.63
Age (years)	30 ± 11	31 ± 12	0.31
Height (cm)	174 ± 9	174 ± 8	0.36
Weight (kg)	72 ± 13	73 ± 13	0.67
BMI (kg/m^2)	23.8 ± 3.5	23.7 ± 3.5	0.61
Days between injury and reconstruction	248 ± 472	430 ± 1065	0.06
Type of graft			
Hamstrings	269 (80.5%)	129 (82.7%)	0.26
Patella	56 (16.8%)	25 (16.0%)	
Allograft	1 (0.3%)	2 (1.3%)	
Quadriceps	2 (0.6%)	0 (0%)	
Other	6 (1.8%)	0 (0%)	

*ACL* anterior cruciate ligament, *n* number of patients, *cm* centimeters, *kg* kilograms, *m* meters, *BMI* body mass index, *HV* high-volume clinics, *LV* low-volume clinics

For categorical variables, *n* (%) is presented

For continuous variables, the mean ± SD is presented

**Fig. 4:**
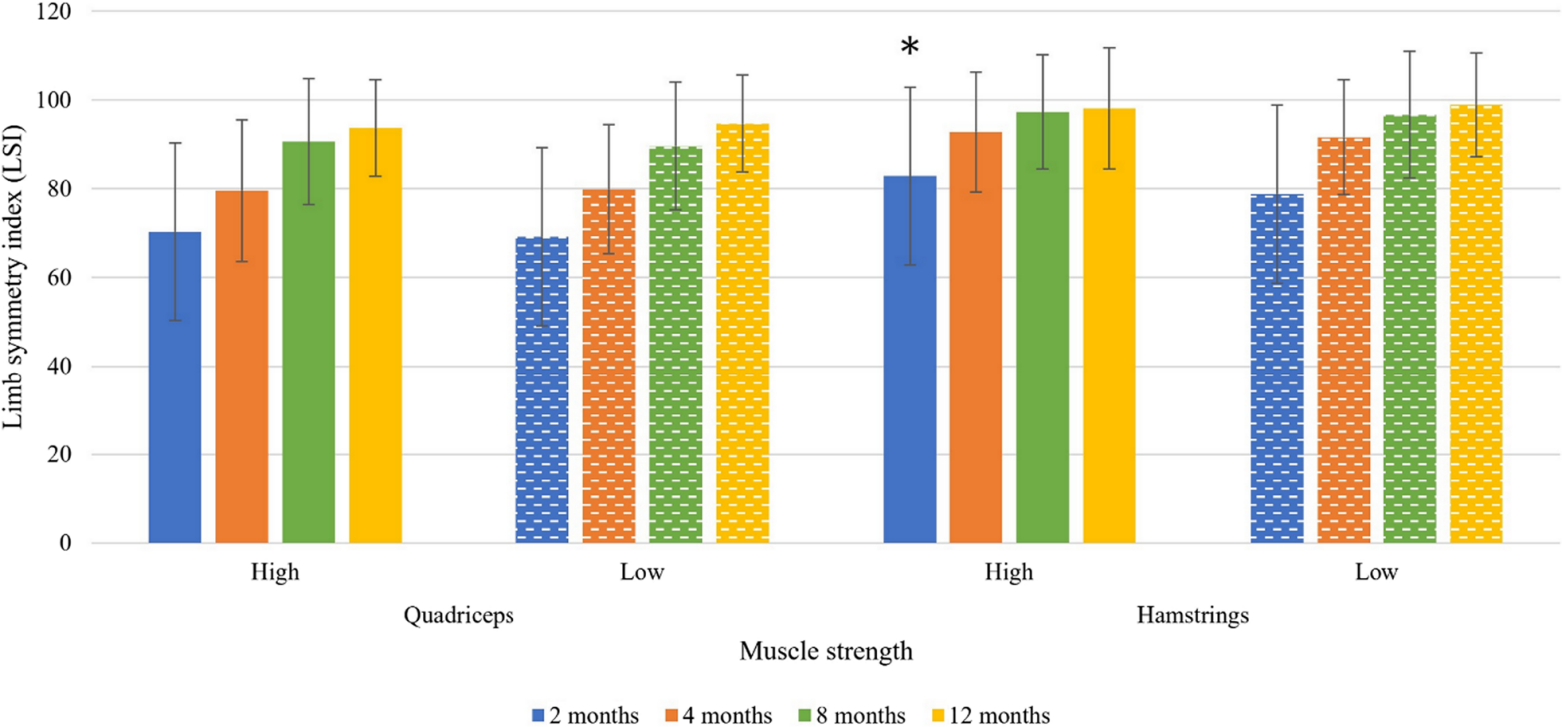
Solid and dashed bars represent the high and low volume clinics, respectively. Whiskers of each bar represent the standard deviation. Muscle strength tests during the first 12 months of rehabilitation after anterior cruciate ligament reconstruction

Patients treated at HV clinics presented more symmetrical hamstring strength at 2 months follow-up, although a small effect size (83.3% ± 19.5% HV vs. 78.4% ± 21.0% LV, *p* = 0.014, *d* = 0.248) and greater passing rates (≥ 90%) for quadriceps strength at 4 months follow-up (33.4% HV vs. 24.5% LV, *p* = 0.048) compared with patients treated at an LV clinic (Fig. 4 and Table 6). There were no other differences in the strength symmetry or passing rates during the first year after ACL reconstruction.

**Table 6:** Muscle strength in the quadriceps and hamstrings muscle groups, first 12 months after ACL-reconstruction

Muscle test	Grou*p*	n	2 months			4 months			8 months			12 months		
			LSI ± SD%	Pass, *n* (%)	*p*-value LSI/Pass	LSI ± SD%	Pass, *n* (%)	*p*-value LSI/Pass	LSI ± SD%	Pass, *n* (%)	*p*-value LSI/Pass	LSI ± SD%	Pass, *n* (%)	*p*-value LSI/Pass
Quadriceps strength	HV	338	70.5 ± 20.0	37 (10.9)	0.55/0.42	79.8 ± 15.7	113 (33.4)	0.95/0.05*	91.0 ± 13.9	201 (59.5)	0.33/1.00	93.8 ± 10.6	232 (68.6)	0.48/0.35
	LV	159	69.3 ± 17.5	13 (8.2)		79.7 ± 14.7	39 (24.5)		89.5 ± 14.7	94 (59.1)		94.7 ± 11.1	116 (73.0)	
Hamstrings strength	HV	338	83.3 ± 19.5	118 (34.9)	0.01*/0.76	92.9 ± 13.5	204 (60.4)	0.24/0.92	97.4 ± 12.9	253 (74.9)	0.38/0.83	97.8 ± 13.5	274 (81.1)	0.45/0.55
	LV	159	78.4 ± 21.0	53 (33.3)		91.3 ± 12.7	95 (59.7)		96.2 ± 14.4	117 (73.6)		98.8 ± 11.7	125 (78.6)	

*LSI* limb symmetry index, *SD* standard deviation, *HV* high-volume clinic, *LV* low-volume clinic, *n* number of patients, Pass = LSI ≥ 90%,

*Significant (*p* < 0.05) difference between high- and low-volume clinics

For categorical variables, *n* (%) is presented

For continuous variables, the mean ± SD is presented

### Muscle function and patient-reported outcomes

The number of patients who had data from muscle strength tests was 497, while 757 had data from PROs; thus, 328 patients had data from both muscle function and PROs at all follow-up visits. Out of the 328 patients, 70% and 30% were in the HV and LV groups, respectively (Table 7). There were no differences in demographics between HV and LV clinics.

**Table 7:** Demographics for muscle function and PROs for high- and low-volume clinics, first 12 months after ACL-reconstruction

	HV	LV	*p*-value
*n*	231	97	
Women, *n* (%)	120 (51.9%)	51 (52.6%)	1.0
Age (years)	29 ± 10	30 ± 11	0.37
Height (cm)	174 ± 9	175 ± 8	0.22
Weight (kg)	72 ± 14	73 ± 13	0.48
BMI (kg/m^2^)	23.7 ± 3.6	23.7 ± 2.9	0.98
Days between injury and reconstruction	240 ± 423	407 ± 1080	0.14
Type of graft			
Hamstrings	187(81.7%)	78 (83.0%)	0.62
Patella	39 (17.0%)	15 (16.0%)	
Allograft	0 (0%)	1 (1.1%)	
Quadriceps	1 (0.4%)	0 (0%)	
Other	2 (0.9%)	0 (0%)	

*ACL* anterior cruciate ligament, *n* number of patients, *cm* centimeters, *kg* kilogram, *BMI* body mass index, *m* meters, *HV* high-volume clinic, *LV* low-volume clinic

For categorical variables, *n* (%) is presented

For continuous variables, the mean ± SD is presented

There were no significant differences between patients treated at HV clinics and those treated at LV clinics in LSI or passing rates for muscle function tests at any follow-up. Limb symmetry index and passing rates for all muscle function tests are presented in Additional files 6 and 7. Table 8 presents the differences in PROs at follow-up between the HV and LV groups for patients with data from both muscle function tests and PROs at all follow-up visits during the first 12 months after ACL reconstruction. The HV group presented significantly greater K-SES_18_ at 2 months follow-up (4.5 ± 1.8 HV vs. 3.9 ± 1.7 LV, *p* = 0.01, *d* = 0.30), KOOS-Sports at 2 months follow-up (34.1 ± 22.0 HV vs. 27.2 ± 20.4 LV, *p* = 0.01, *d* = 0.32), and ACL-RSI at 12 months follow-up (77.7 ± 29.2 HV vs. 69.3 ± 32.9 LV, *p* = 0.02, *d* = 0.28), all results had a small effect size.

**Table 8:** Patient-reported outcomes for high- and low-volume clinics, all follow-ups, first 12 months after ACL-reconstruction

PROs	Group	2 months	4 months	8 months	12 months
K-SES_18_ present	HV	4.5 ± 1.8*	6.3 ± 1.7	8.2 ± 1.2	8.9 ± 1.1
	LV	3.9 ± 1.7	6.0 ± 1.6	8.0 ± 1.1	8.8 ± 1.1
K-SES_18_ future	HV	7.6 ± 1.6	7.6 ± 1.6	7.8 ± 1.6	7.9 ± 1.6
	LV	7.5 ± 1.6	7.7 ± 1.4	7.9 ± 1.2	7.9 ± 1.4
KOOS-pain	HV	79.9 ± 12.2	84.2 ± 10.1	88.9 ± 9.1	90.5 ± 10.4
	LV	78.1 ± 12.3	83.5 ± 10.6	87.7. ± 10.4	90.2 ± 9.8
KOOS-symptom	HV	67.2 ± 15.9	74.9 ± 14.5	80.7 ± 14.4	83.6 ± 13.5
	LV	66.4 ± 16.6	75.0 ± 14.3	79.1 ± 16.3	83.3 ± 15.5
KOOS-sports	HV	34.1 ± 22.0*	52.8 ± 21.9	72.7 ± 17.7	80.9 ± 17.2
	LV	27.2 ± 20.4	51.8 ± 20.6	70.9 ± 16.0	78.6 ± 17.8
KOOS-quality	HV	40.4 ± 16.2	48.1 ± 16.3	58.5 ± 17.3	67.2 ± 16.3
	LV	37.7 ± 16.6	46.9 ± 14.8	57.9 ± 16.3	65.3 ± 18.1
ACL-RSI	HV			66.7 ± 30.0	77.7 ± 29.2*
	LV			59.6 ± 31.9	69.3 ± 32.9

*PROs* patient-reported outcomes, *K-SES*_*18*_ knee self-efficacy scale, *KOOS* knee injury and osteoarthritis outcome score, *ACL-RSI* anterior cruciate ligament return to sports index, *HV* high-volume clinic, *LV* low-volume clinic

*Significant (*p* < 0.05) difference between HV and LV clinics. Results are presented as mean ± SD

## Discussion

Our main findings were that no clinically relevant differences were found between PT clinics with high- and low-patient volumes in terms of the incidence of a second ACL injury, rate of return to pre-injury level of activity, rate of passing RTS tests, or in the recovery of muscle function tests, or the results of the PROs during the first year after ACL reconstruction. Despite a few early significant findings, the effect sizes for differences were small and, therefore, considered to be of no clinical relevance.

An increased understanding of how patient volume influences outcomes during rehabilitation after ACL reconstruction is important, especially with regard to the reported increased incidence of ACL injuries [24]. Previous studies have mainly concentrated on the influence of hospital volume on surgical outcomes [13, 25–27]. It seems reasonable to assume that high patient volumes develop clinical experience and expertise among surgeons, favoring outcomes in high-volume clinics. Although research on high versus low-volume clinics with respect to rehabilitation clinics is limited, it is tempting to generalize findings from previous research on volume effects on rehabilitation. However, ACL rehabilitation is complex to measure as it is individualized, with different rehabilitation phases in which patients face both psychological [28] and physical challenges [29] over a considerable time (commonly > 9 months) [30]. Based on our results, clinical volumes do not appear to be directly related to rehabilitation outcomes during the first or second year of rehabilitation or to the odds of a second ACL injury.

### Second ACL injuries

The crude second ACL injury rate, defined as re-injury or contralateral ACL injury, in the cohort in this study was 1.5% for HV clinics and 1.6% for LV clinics within 1 year (OR 0.95) after ACL reconstruction and within 2 years 4.4% for HV clinics, and 3.8% for LV clinics (OR 1.13). Rates of second ACL injuries were lower than those reported by Wiggins et al. [5], 23% in patients younger than 25 years who underwent RTS. However, the analysis by Wiggins et al. [5] was performed on a younger cohort with a longer follow-up time. Previous research has also shown that returning to higher levels of sports is associated with a greater risk of sustaining a second ACL injury [31]. In the cohort included in this study, the rate of return to knee-strenuous sports, Tegner level ≥ 6, was 26–31% at 12 months follow-up, which is in line with previous research [32]. This, in turn, may partly explain the relatively low risk for a second ACL injury in our study, since almost two out of three patients had not yet returned to their pre-injury level of activity after ACL reconstruction. The results presented here concerning the incidence of a second ACL injury with respect to rehabilitation clinic patient volume, suggest that patient volume does not directly affect the odds for a second ACL injury, at least not if patients are continuously assessed with tests of muscle function and PROs. In this study, we could not determine whether the clinical experience of the individual PTs might be more important than the experience of a rehabilitation clinic. The individual experience of a PT can be equal in LV and HV clinics. However, it is reasonable to assume that the collective experience is higher at HV clinics compared to LV clinics, and patients with more severe injuries might be more prone to seek help from HV clinics. While the individual clinical experience of the treating PTs is not known and cannot be determined by this study, the experience level of each PT included in Project ACL might be one reason why our results did not show significant differences between clinics. Interestingly, continuous follow-ups throughout rehabilitation and special education for clinicians participating in the rehabilitation registry, Project ACL, can potentially have provided PT with a greater insight into the patient’s readiness for sport and the risk associated with activities such as cutting and pivoting, and therefore facilitate decisions on gradual return to such exposure with respect to the patient’s recovery.

Treating patients with ACL injury is a complex matter involving both physical and psychological aspects, which can ultimately provide PTs with important clinical experience. Therefore, it is yet to be determined whether there is a learning curve for the individual PT with regard to postoperative outcomes after ACL reconstruction, and whether individual experience can influence outcomes for patients after ACL reconstruction better than clinical volume. It is also necessary to consider whether the tests used in RTS clearance are insensitive to differences.

### Return to pre-injury level of activity and patient reported outcomes

Approximately one in three patients had returned to their pre-injury level of sports (Tegner pre-injury ≥ 6) at the 12 months follow-up, regardless of clinical status. Previous research has reported that approximately 50% of patients participating in jumping, pivoting, and cutting sports return to the pre-injury level of activity 1 year after ACL reconstruction [33]. Whether the lower rate of return to pre-injury level of activity (Tegner pre-injury ≥ 6) in our study is a result of a short follow-up time or reflecting an unreadiness to return to sports within 1 year after ACL reconstruction is unknown. Fear of re-injury has been reported as the main reason for not returning to the pre-injury sports level after ACL reconstruction [34]. Ardern et al. [3] reported that two of five patients may be able to return within 2 years. Consequently, it is possible that the patients in our study may need a longer period of rehabilitation than 1 year before returning to the pre-injury level of activity after ACL reconstruction. More encouraging, 62–71% of patients with a lower pre-injury level of activity (Tegner 1–5) returned to the same pre-injury level within 1 year after ACL reconstruction. In conclusion, HV clinics have a greater proportion of patients returning to pre-injury sports levels at 2 and 4 months in the subgroup of patients active on a Tegner level of 1–5 (34% vs. 11%, and 42% vs. 23%, respectively). However, at the 1-year follow-up, there were no significant differences between the HV and LV clinics in return to the pre-injury level of sports, regardless of Tegner pre-injury.

### Muscle function

Regardless of whether patients underwent rehabilitation at a high- or low-volume clinic, patients who had attended every follow-up for strength testing during the first year of rehabilitation on average demonstrated an LSI ≥ 90% in hamstring strength at the 4 months follow-up, and LSI ≥ 90% in quadriceps strength at 1 year follow-up. Recovering symmetrical muscle function (LSI ≥ 90%) can protect against the risk of a second ACL injury [35–40]. Our cohort presented superior results in terms of recovering symmetrical quadriceps strength at the 1 year follow-up (68.6% for HV and 73.0% for LV) compared with previous research (43.5% [41, 42]), which may be explained by participation in the rehabilitation registry, Project ACL, which continuously provides patients and caregivers with feedback regarding muscle strength and function. The high passing rates of LSI might be one of the reasons why patients in our cohort presented lower rates of re-injury than those in previous studies [5]. Furthermore, the feedback provided from regular assessments with muscle function tests and PROs might give the patient and the responsible PT a better understanding of readiness for RTS and might therefore also delay RTS to after one year of rehabilitation as a risk reduction procedure. Feedback can also provide guidance in the rehabilitation process, which may explain the finding of similar outcomes between HV and LV clinics in our study. However, the feedback available to all clinicians registered in the Project ACL may limit the generalizability of our results, as feedback from standardized and regular assessments is not always available at PT clinics, as is the case in Project ACL.

### Limitations

One limitation of our study was that over half of the total number of patients registered in the Project ACL did not report which clinic they attended for rehabilitation; thus, these patients were excluded. Since the analysis are performed for patients who have muscle function tests or PROs registered from all follow-ups, this might risk to an attention bias, i.e., analyses might only investigate compliant at respective clinic. Furthermore, the patients, their PTs or orthopedic surgeon manually register the presence of a new ACL injury in the database, implying a risk of unreported ACL injuries in the database. To mitigate this risk we used a compliant cohort for this study. Second, concomitant injuries such as cartilage or meniscal injuries are thought to prolong rehabilitation, limit the likelihood of returning to the pre-injury level of activity, and increase the risk of a second ACL injury [43, 44]. Currently, data on concomitant injuries are not collected in the registry, therefore, we cannot determine whether concomitant injuries have affected our results. Third, some patients changed clinics during the rehabilitation process, either from a high-volume clinic to a low-volume clinic, or vice versa. To address this limitation, we used the clinic that the patient had attended at most follow-ups to determine if the patient was grouped in a high- or low-volume clinic. Furthermore, data from patients who had available data from all follow-ups during the first year were extracted for the outcomes of interest. Working at a high-volume clinic does not necessarily mean that the PT is more experienced than the PT at a low-volume clinic. It should also be noted that our analysis was based on clinical volume and not on the specific PTs patient volume or years of experience. Another potential proxy that may reflect PT experience, is the number of visits patients attend during their rehabilitation and what type of rehabilitation the patients perform during this time. However, PTs who are connected to Project ACL are both from HV and LV clinics and are provided with the same amount of feedback and educational programs, where the ambition to spread knowledge through ﻿Project ACL may have been largely successful, which can potentially explain the lack of significant differences in outcomes between HV and LV clinics. Perhaps Project ACL itself should be considered as a “large volume clinic,” where PTs work in very similar ways, albeit in many different commercial clinics. Project ACL can function as a specialized sports medicine center with a more progressive rehabilitation model for tackling the challenge that patients must go to an HV clinic without having the possibility to and can therefore go to an LV clinic connected to Project ACL and obtain the same quality outcome results. On this topic, the difference in cases seeking medical care between the clinic volumes can affect the results, whereas more severe knee cases of knee injury probably seek assistance with their rehabilitation at a high-volume clinic, which might equalize results, although it has not been possible to confirm this from this study. Furthermore, previous studies have showed that surgical volumes can affect outcomes after knee surgery, and that clinic and the surgeons experience for ACL reconstruction has not been considered in this study. Finally, due to the large number of analyses performed in our study, in combination with a significance level of 0.05, there is a risk of type-1 errors. To address this, significant findings were interpreted together with Cohen´s d to evaluate the magnitude of significance.

### Future research

To better understand the effect of patient volume on rehabilitation outcomes after ACL reconstruction, future research should first compare cross-sectional data between patients in the HV and LV clinics. Second, it would be interesting to investigate individual experience or patient satisfaction with the unique PT, how this experience influences the rehabilitation outcomes for the patients, and whether there is a learning curve which a senior, experienced, PT has passed making their treatment more effective. Third, we intend to explore the reasons why patients choose to change rehabilitation clinics, and whether the lack of differences between HV and LV clinics persists at long-term follow-up.

## Conclusions

No clinically relevant difference in the incidence of secondary ACL injuries in patients who underwent rehabilitation after ACL reconstruction at high- or low-volume PT clinics was found. In addition, no clinically relevant differences in outcomes were found during the first year in terms of patient-reported outcomes, recovery of muscle function, or return to pre-injury activity.

### Supplementary Information

**Additional file 1**. Tests performed at scheduled follow-ups in Project ACL.

**Additional file 2**. Patients per clinic registered in Project ACL at the time of data extraction.

**Additional file 3**. Second ACL injury during the first year stratified by age.

**Additional file 4**. Second ACL injury during the first 2 years stratified by age.

**Additional file 5**. Patient reported outcomes during the first year after anterior cruciate ligament reconstruction.

**Additional file 6**. Prospective muscle function test during the first year of rehabilitation after ACL reconstruction.

**Additional file 7**. Prospective muscle function test during the first year of rehabilitation after ACL reconstruction.

## Data Availability

The datasets used and/or analyzed during the current study are available from the corresponding author on reasonable request.

## Abbreviations

ACL: Anterior cruciate ligament
ACL-RSI: Anterior cruciate ligament-return to sport after injury
CI: Confidence interval
cm: Centimeters
*d*: Cohens *d*′
HV: High volume
K-SES_18_: Knee-Self Efficacy Scale_18_
KOOS: Knee injury and osteoarthritis outcome score
LSI: Limb symmetry index
LV: Low volume
Min–max: Minimum–maximum
Nm: Newtonmeters
OR: Odds ratio
PROs: Patient reported outcomes
RECORD: REporting of studies Conducted using Observational Routinely Collected Health Data
RTS: Return to sports
SD: Standard deviation
Tegner: Tegner activity scale
