# Supplementary material for: A comparison between physical therapy clinics with high and low rehabilitation volumes of patients with ACL reconstruction
Source: J Orthop Surg Res. 2023 Nov 7;18:842. doi: 10.1186/s13018-023-04304-4 (PMC10629052; doi:10.1186/s13018-023-04304-4)
Supplement: Supplementary file 1 — Additional file 1. Tests performed at scheduled follow-ups in Project ACL. [file 13018_2023_4304_MOESM1_ESM.docx]

| **Additional file 1. Tests performed at scheduled follow-ups in Project ACL.** | | | | | | |
| --- | --- | --- | --- | --- | --- | --- |
| Follow-up | Muscle strength tests | Hop tests | Tegner Activity Scale | Knee injury and Osteoarthritis Outcome Score | Knee Self-efficacy Scale | Anterior Cruciate Ligament-Return to Sport after Injury scale |
| 2 months | X |  | X | X | X |  |
| 4 months | X | X | X | X | X |  |
| 8 months | X | X | X | X | X | X |
| 12 months | X | X | X | X | X | X |
| 18 months | X | X | X | X | X | X |
| 24 months | X | X | X | X | X | X |
| 5 years | X | X | X | X | X | X |
| Every 5^th^ year | X | X | X | X | X | X |
