# Supplementary figures and images for: A comparison between physical therapy clinics with high and low rehabilitation volumes of patients with ACL reconstruction
Source: J Orthop Surg Res. 2023 Nov 7;18:842. doi: 10.1186/s13018-023-04304-4 (PMC10629052; doi:10.1186/s13018-023-04304-4)

**Additional file 2.** Patients per clinic registered in Project ACL at the time of data extraction.

**
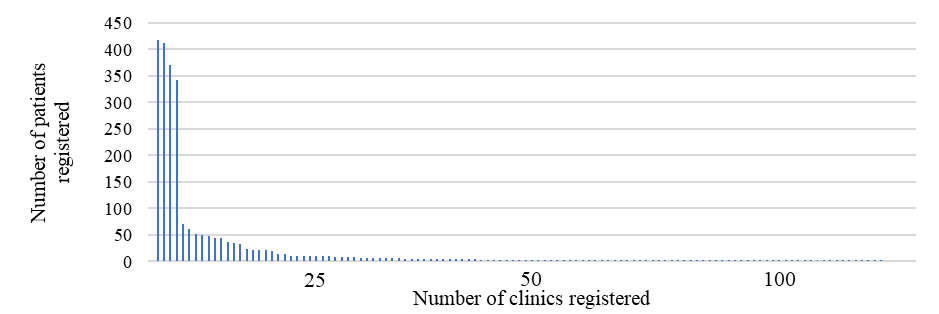
**

Supplement: Supplementary file 2 — Additional file 2. Patients per clinic registered in Project ACL at the time of data extraction. [file 13018_2023_4304_MOESM2_ESM.docx]

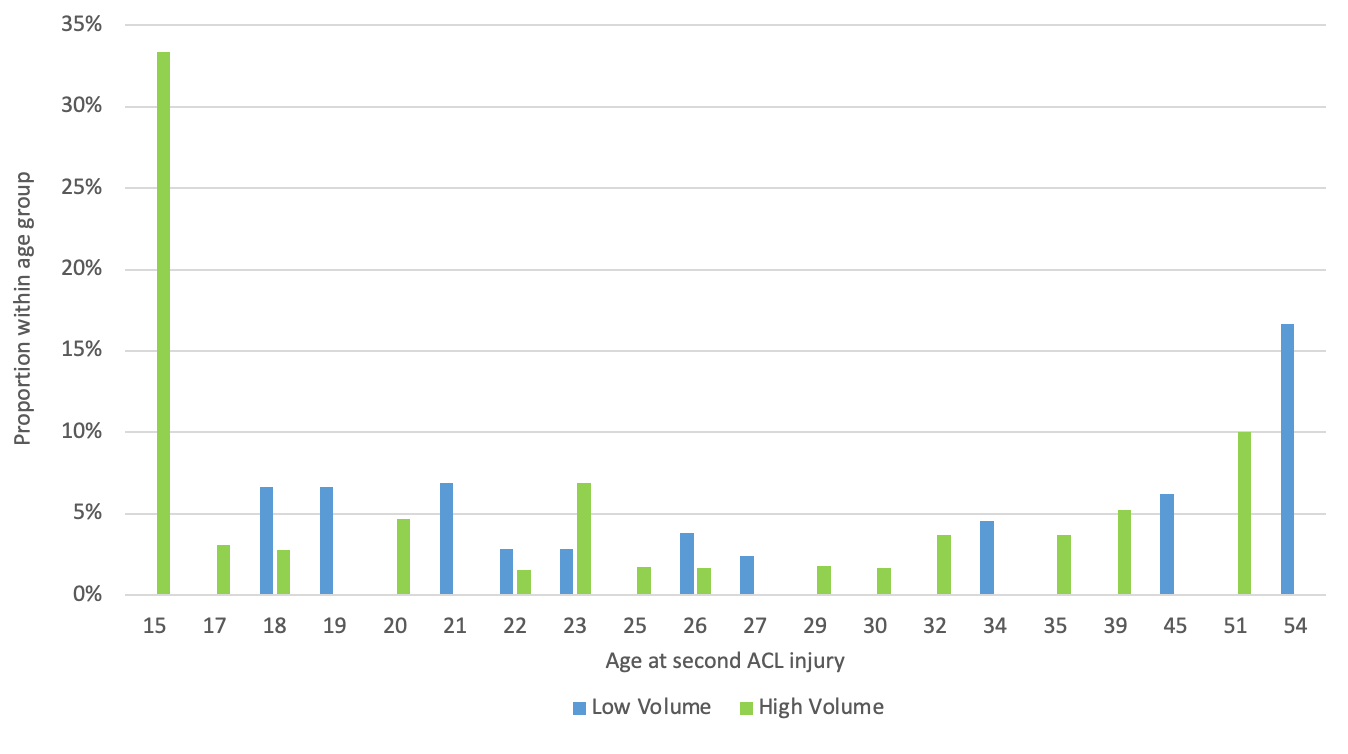


**Appendix Figure 1.** Second ACL injury during the first year stratified by age.

Supplement: Supplementary file 3 — Additional file 3. Second ACL injury during the first year stratified by age. [file 13018_2023_4304_MOESM3_ESM.docx]

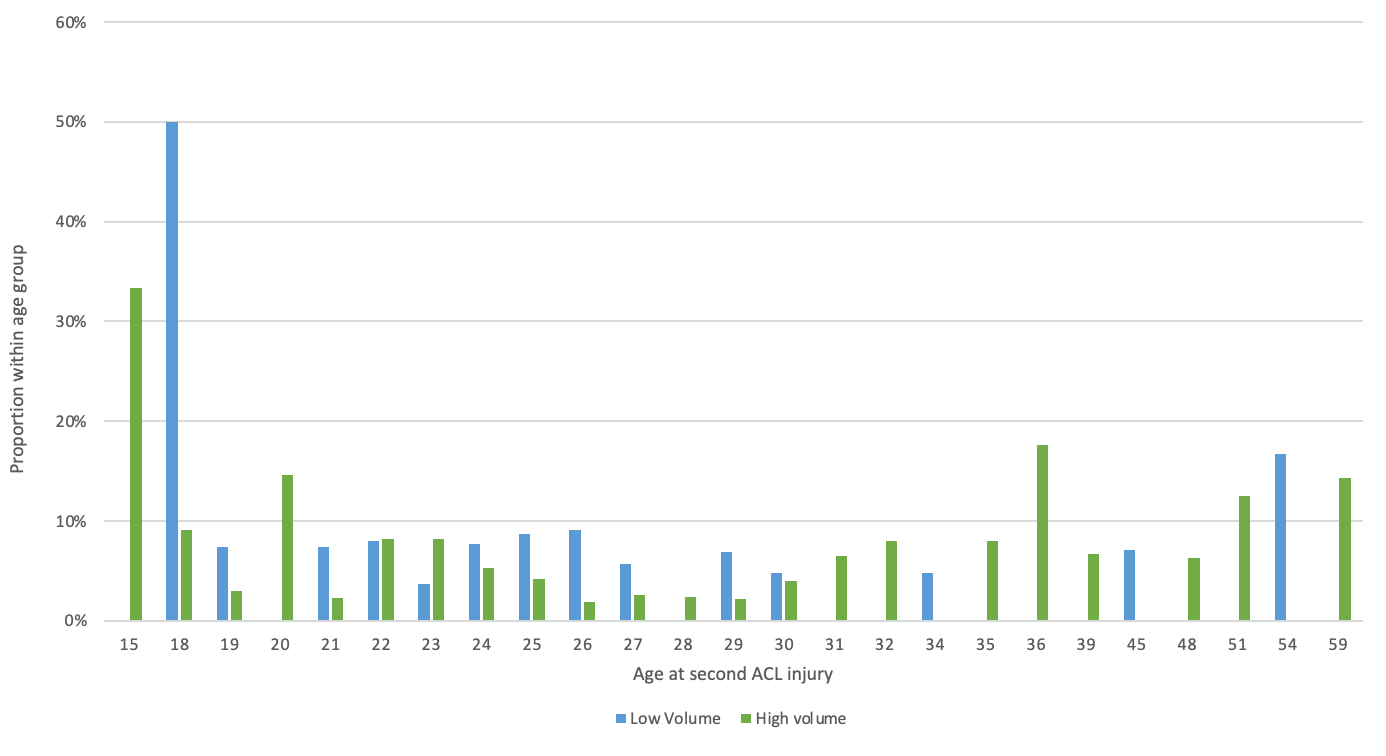
**Appendix Figure 2.** Second ACL injury during the first 2 years stratified by age.

Supplement: Supplementary file 4 — Additional file 4. Second ACL injury during the first 2 years stratified by age. [file 13018_2023_4304_MOESM4_ESM.docx]
