# Supplementary material for: A comparison between physical therapy clinics with high and low rehabilitation volumes of patients with ACL reconstruction
Source: J Orthop Surg Res. 2023 Nov 7;18:842. doi: 10.1186/s13018-023-04304-4 (PMC10629052; doi:10.1186/s13018-023-04304-4)
Supplement: Supplementary file 5 — Additional file 5. Patient reported outcomes during the first year after anterior cruciate ligament reconstruction. [file 13018_2023_4304_MOESM5_ESM.docx]

| Appendix Table 1: Patient reported outcomes during the first year after anterior cruciate ligament reconstruction | | | | | |  |
| --- | --- | --- | --- | --- | --- | --- |
| PROs | Group | 2 months | 4 months | 8 months | 12 months | |
| K-SES Present | HV | 4.2 ± 1.9 | 5.8 ± 1.9 | 7.6 ± 1.7 | 8.3 ± 1.6 | |
|  | LV | 3.9 ± 1.9 | 5.7 ± 1.9 | 7.5 ± 1.7 | 8.3 ± 1.5 | |
| K-SES Future | HV | 7.3 ± 1.7 | 7.2 ± 1.6 | 7.3 ± 1.8 | 7.3 ± 1.9 | |
|  | LV | 7.4 ± 1.6 | 7.5 ± 1.6 | 7.3 ± 1.8 | 7.4 ± 1.8 | |
| ACL-RSI | HV |  |  | 59.9 ± 30.4 | 69.9 ± 30.6 | |
|  | LV |  |  | 59.1 ± 29.1 | 67.9 ± 29.5 | |

PROs, Patient-reported Outcomes; K-SES, Knee Self-Efficacy Scale; ACL-RSI, Anterior Cruciate Ligament-Return to Sports After Injury Scale; HV, High Volume Clinics; LV, Low Volume Clinics
