# Supplementary material for: A comparison between physical therapy clinics with high and low rehabilitation volumes of patients with ACL reconstruction
Source: J Orthop Surg Res. 2023 Nov 7;18:842. doi: 10.1186/s13018-023-04304-4 (PMC10629052; doi:10.1186/s13018-023-04304-4)
Supplement: Supplementary file 6 — Additional file 6. Prospective muscle function test during the first year of rehabilitation after ACL reconstruction. [file 13018_2023_4304_MOESM6_ESM.docx]

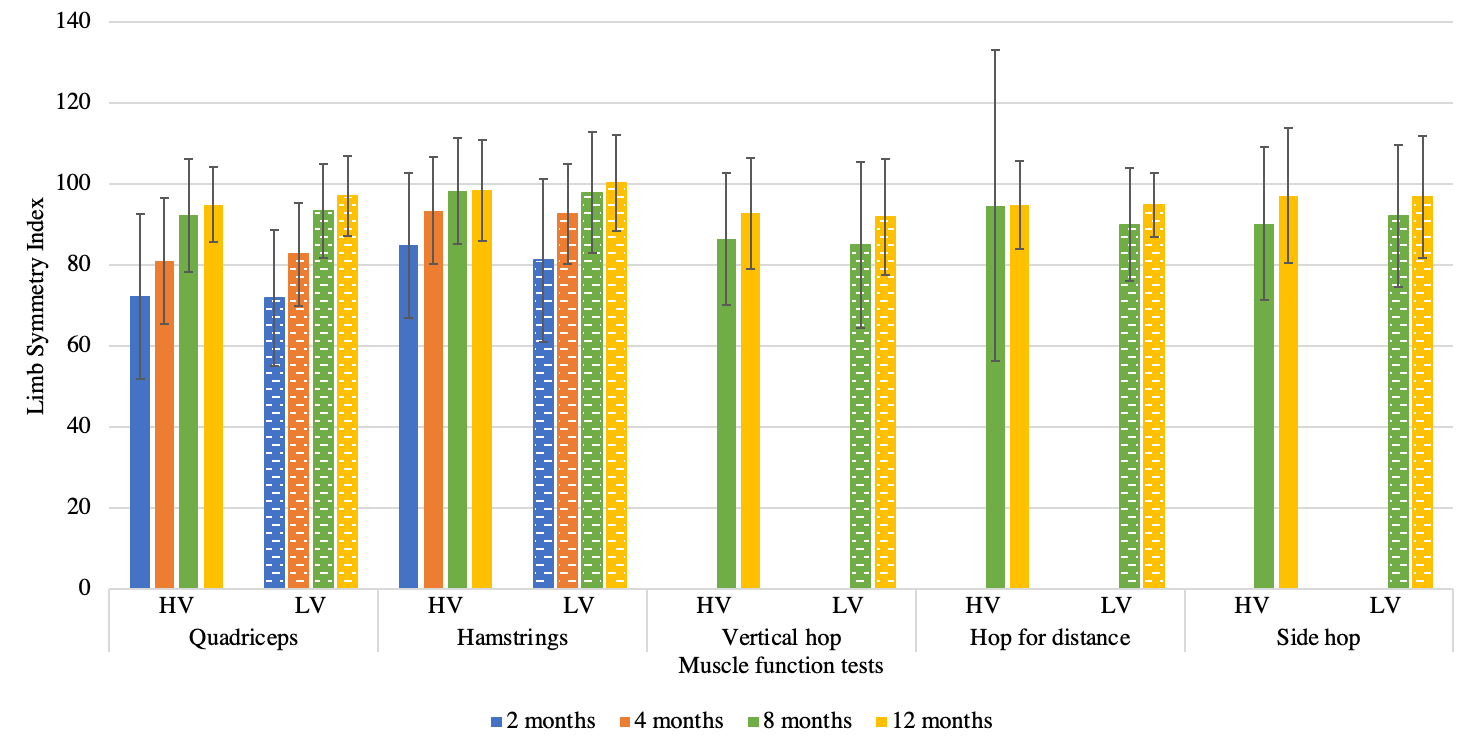
**Appendix Figure 3.** Prospective muscle function test during the first year of rehabilitation after ACL reconstruction. Solid bars represent high-volume clinics and dashed bars represent low-volume clinics. Standard deviation is presented for each bar with whiskers. HV, high-volume clinics; LV, low-volume clinics.
