# Supplementary material for: A comparison between physical therapy clinics with high and low rehabilitation volumes of patients with ACL reconstruction
Source: J Orthop Surg Res. 2023 Nov 7;18:842. doi: 10.1186/s13018-023-04304-4 (PMC10629052; doi:10.1186/s13018-023-04304-4)
Supplement: Supplementary file 7 — Additional file 7. Prospective muscle function test during the first year of rehabilitation after ACL reconstruction. [file 13018_2023_4304_MOESM7_ESM.docx]

| **Appendix Table 2:** Prospective muscle function test during the first year of rehabilitation after ACL reconstruction. | | | | | | | | | | | | | | |
| --- | --- | --- | --- | --- | --- | --- | --- | --- | --- | --- | --- | --- | --- | --- |
|  |  |  | 2 months | | | 4 months | | | 8 months | | | 12 months | | |
| Muscle test | Group | n | LSI ± SD% | Pass, n(%) | p-value LSI/Pass | LSI ± SD% | Pass, n(%) | p-value LSI/Pass | LSI ± SD% | Pass, n(%) | p-value LSI/Pass | LSI ± SD% | Pass, n(%) | p-value LSI/Pass |
| Quadriceps strength | HV | 231 | 72.3± 20.4 | 30 (13.0) | 0.88/0.58 | 81.1± 15.6 | 89 (38.5) | 0.33/0.08 | 92.3± 13.9 | 149 (64.5) | 0.45/0.45 | 95.0± 9.2 | 174 (75.3) | 0.06/0.19 |
|  | LV | 97 | 71.9± 16.9 | 10 (10.3) |  | 82.7± 12.7 | 27 (27.8) |  | 93.5± 11.6 | 67 (69.1) |  | 97.2± 10.0 | 80 (82.5) |  |
| Hamstrings strength | HV | 231 | 85.0± 18.0 | 88 (38,1) | 0.10/0.62 | 93.5± 13.2 | 149 (64.5) | 0.54/0.71 | 98.3± 13.1 | 178 (77.1) | 0.87/0.56 | 98.5± 12.4 | 188 (81.4) | 0.21/0.75 |
|  | LV | 97 | 81.2± 20.0 | 34 (35.1) |  | 92.6± 12.4 | 60 (61.9) |  | 98.0± 15.0 | 78 (80.4) |  | 100.3± 11.9 | 81 (83.5) |  |
| Vertical hop | HV | 231 |  |  |  |  |  |  | 86.5± 16.3 | 105 (45.5) | 0.54/0.81 | 92.8± 13.7 | 148 (64.1) | 0.60/0.17 |
|  | LV | 97 |  |  |  |  |  |  | 85.0± 20.4 | 42 (43.3) |  | 91.9± 14.4 | 54 (55.7) |  |
| Hop for distance | HV | 231 |  |  |  |  |  |  | 94.7± 38.4 | 159 (68.8) | 0.24/0.20 | 94.9± 11.0 | 180 (77.9) | 0.91/0.55 |
|  | LV | 97 |  |  |  |  |  |  | 90.0± 14.0 | 59 (60.8) |  | 94.8± 7.9 | 79 (81.4) |  |
| Side hop | HV | 231 |  |  |  |  |  |  | 90.3± 19.0 | 136 (58.9) | 0.39/0.81 | 97.2± 16.7 | 174 (75.3) | 0.90/0.68 |
|  | LV | 97 |  |  |  |  |  |  | 92.2± 17.6 | 59 (60.8) |  | 97.0± 15.0 | 71 (73.2) |  |

n, number of patients; LSI, limb symmetry index; SD, standard deviation; pass, ≥90% LSI.
